# Molecular Characterization of Cultivated Bromeliad Accessions with Inter-Simple Sequence Repeat (ISSR) Markers

**DOI:** 10.3390/ijms13056040

**Published:** 2012-05-18

**Authors:** Fei Zhang, Yaying Ge, Weiyong Wang, Xinying Yu, Xiaolan Shen, Jianxin Liu, Xiaojing Liu, Danqing Tian, Fuquan Shen, Yongming Yu

**Affiliations:** Flower Research and Development Centre, Zhejiang Academy of Agricutural Sciences, Hangzhou 311202, China; E-Mails: gyy954002@126.com (Y.G.); hwwy@xs.hz.zj.cn (W.W.); yxy620821@163.com (X.Y.); angalla@163.com (X.S.); ljxljx20002000@yahoo.com.cn (J.L.); liuxiaojingcau@126.com (X.L.); tdq0123@163.com (D.T.); SFQUAN@sina.com (F.S.); yuyongm@163.com (Y.Y.)

**Keywords:** cultivated bromeliads, genetic diversity, population structure, ISSR

## Abstract

Bromeliads are of great economic importance in flower production; however little information is available with respect to genetic characterization of cultivated bromeliads thus far. In the present study, a selection of cultivated bromeliads was characterized via inter-simple sequence repeat (ISSR) markers with an emphasis on genetic diversity and population structure. Twelve ISSR primers produced 342 bands, of which 287 (~84%) were polymorphic, with polymorphic bands per primer ranging from 17 to 34. The Jaccard’s similarity ranged from 0.08 to 0.89 and averaged ~0.30 for the investigated bromeliads. The Bayesian-based approach, together with the un-weighted paired group method with arithmetic average (UPGMA)-based clustering and the principal coordinate analysis (PCoA), distinctly grouped the bromeliads from *Neoregelia*, *Guzmania*, and *Vriesea* into three separately clusters, well corresponding with their botanical classifications; whereas the bromeliads of *Aechmea* other than the recently selected hybrids were not well assigned to a cluster. Additionally, ISSR marker was proven efficient for the identification of hybrids and bud sports of cultivated bromeliads. The findings achieved herein will further our knowledge about the genetic variability within cultivated bromeliads and therefore facilitate breeding for new varieties of cultivated bromeliads in future as well.

## 1. Introduction

Bromeliads (Bromeliaceae) mainly originate from subtropical and tropical America. Bromeliaceae comprise about 56 genera and 3000 species [1]. Due to their attractive characteristics regarding regular plant architecture, various flower types and colors, bromeliads specific to such genera as *Guzmania*, *Vriesea*, *Neoregelia*, and *Aechmea* presently comprise the most economically important ornamentals worldwide. Currently, the demand for ornamental bromeliads increases year after year. In China, large-scale commercial production of bromeliads started in the 1990s, and now bromeliads together with orchids and anthuriums have become the top three potted flowers. However, the current commercially cultivated bromeliads in China were mainly introduced from overseas, due to the lack of bromeliad germplasm. During the past decade, a large number of bromeliads have been collected throughout the world. This allows for the possibility of breeding new bromeliad varieties to break the bottleneck of Chinese bromeliad industry. To our knowledge, most of the commercial bromeliad varieties are complex interspecific hybrids of several species, and often their parentages are unknown. Thus complete characterization of the collected bromeliads will be beneficial to the efficient utilization of these germplasm.

So far, previous researchers have focused great efforts on systematics of the Bromeliaceae family [2,3], and also population genetics with respect to the wild species of *Aechmea* [4,5], *Alcantarea* [6,7], *Tillandsia* [8], *Vriesea* [9,10], *Encholirium* [11] and *Puya* [12]. Nevertheless, genetic diversity in the gene pool of commercially cultivated bromeliads has been scarcely investigated until now. Zhang *et al.* [13], however, reported the genetic relatedness among *Aechmea* species and hybrids comparatively using pedigree data and AFLP markers.

Inter-simple sequence repeat (ISSR) refers to the amplification of DNA region located between two microsatellites loci [14], and also combines the advantages of RAPD markers with high polymorphism and reliability of microsatellites. This marker type has been successfully employed to construct genetic maps of pineapple (*Ananas comosus*) that belong to Bromeliaceae family [15,16]. Here, the present study was aimed to: (1) evaluate the efficiency of ISSR markers for discriminating cultivated bromeliads; and (2) characterize the cultivated bromeliads with an emphasis on genetic diversity and population structure. Findings of this study will further our knowledge of cultivated bromeliads and also contribute to the design of a sound breeding program in future.

## 2. Materials and Methods

### 2.1. Plant Materials

A total of 40 bromeliad accessions, 6 from *Neoregelia*, 8 from *Guzmania*, 10 from *Vriesea*, and 16 from *Aechmea*, were investigated in this study and cultivated as potted plants in the greenhouse under semi-controlled conditions. Of the investigated bromeliads, 33 were representative varieties for the currently cultivated bromeliads in China, and the accession N4-1 was bud sport of N4 (“Flandria”), and the other six accessions, *i.e.*, S1 to S6, were selected hybrids from A50 × *A. recurvata* var. *recurvata* (Table 1). The investigated accessions are maintained at Flower Research and Development Centre, Zhejiang Academy of Agricultural Sciences, Hangzhou, China.

### 2.2. DNA Extraction

Young leaves were collected from each accession, frozen with liquid nitrogen and grinded into powder. Genomic DNA was extracted following a CTAB-based procedure as Murray and Thompson [17] described. DNA concentration was estimated in comparison with known concentrations of Lamda DNA via 0.8% agarose gel.

### 2.3. ISSR Analysis and Band Scoring

A total of 97 ISSR primers were screened for polymorphism in this study. All PCR amplifications were performed in 10 μL reaction volume containing 1 × PCR buffer, 3 mM Mg^2+^, 200 μM dNTP, 0.5 U *Taq* DNA polymerase (Takara, Dalian, China), 10 μM of each primer and 25 ng DNA template. The amplification regime: an initial denaturing step at 94 °C/5 min, followed by 40 cycles of 94 °C/45 s, 55 °C/45 s and 72 °C/90 s, and a final extension step at 72 °C/10 min. The amplicons of ISSR were electrophoresed through 8% non-denaturing polyacrylamide gels run at 300V for 2.5 h in 0.5 × TBE buffer, and visualized by silver staining [18]. Amplified bands were scored 1/0 as presence/absence of bands of the same size for each primer combination to generate the 0/1-matrix.

### 2.4. Data Analysis

The software PowerMarker v3.25 [19] was used to calculate polymorphism information content (PIC) for each primer. Based on the 0/1-matrix, a cluster analysis was conducted based on Jaccard’s similarity coefficients using unweighted pair group method with arithmetic average (UPGMA) method with the SAHN module of NTSYS-pc 2.2 [20], and bootstrapping analysis was performed using FreeTree [21] with 1000 re-samplings in order to test the reliability of the clusters. The COPH (co-phenetic values) routine and MXCOP modules were used to goodness-of-fit between the cluster analysis and original similarity matrix. The principal coordinated analysis (PCoA) was performed with the modules DCENTER and EIGEN implemented in NTSYS-pc, and the two principal coordinates were used to visualize the dispersion of accessions in a two-dimensional array of eigenvectors. Finally, a Bayesian-based approach implemented in STRUCTURE software version 2.3.3 with admixture [22] was performed to deeply explore the population structure of the investigated bromeliads. Five independent simulations with a burn-in period of 100,000 steps followed by 100,000 Monte Carlo Markov Chain replicates [23] were performed with the number of subpopulations (*K*) varying from 1 to 15. That run with the maximum likelihood was applied to assign the accessions into different subgroups [24] with the membership probabilities threshold ≥ 0.80 as well as the maximum membership probability among subgroups. Those accessions < 0.80 membership probabilities were retained in the admixed group. Due to the distribution of log likelihood L(*K*) often did not show a mode for the true *K*, an *ad hoc* measure Δ*K* proposed by Evanno *et al.* [25] was used to detect the true *K* present in the ISSR marker data.

## 3. Results

### 3.1. ISSR Polymorphism

**Figure 1:**
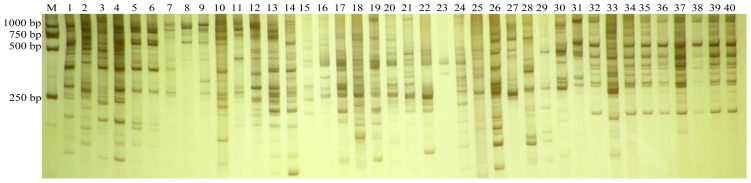
A typical ISSR profile of the 40 accessions of cultivated bromeliads, produced by U848. M indicates DNA marker ladder. The accession number refers to Table 1.

Out of the 97 ISSR primers screened in this study, 12 ISSR primers generating a high level of polymorphism and a clear banding pattern were chosen to genotype the 40 bromeliad accessions. The 12 ISSR primers yielded a total of 342 bands with an average of 28.5 bands per primer. Of the 342 bands, 287 (83.92%) were polymorphic. The number of polymorphic bands detected with each primer varied from 17 (U812) to 34 (U873) and averaged 23.92 (Table 2). The PIC for each individual ISSR primer ranged from 0.26 to 0.37 with a mean value ~0.30, indicating the informativeness of these primers. An typical amplified profile was provided in Figure 1.

### 3.2. Cluster Analysis and PCoA

**Figure 2:**
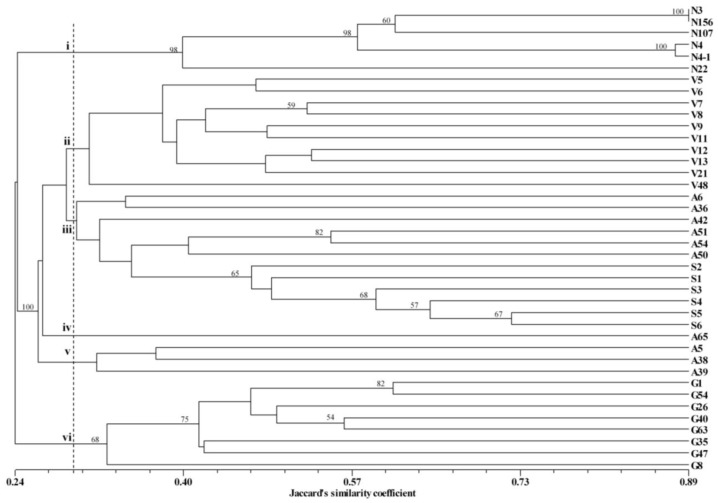
Dendrogram of the cultivated bromeliad accessions, derived from the UPGMA cluster analysis based on Jaccard’s similarity coefficients. The codes refer to Table 1. Numbers on the branches are bootstrap values obtained from 1000 re-samplings, only the bootstrap values > 50% were provided.

Based on the ISSR marker, the Jaccard’s similarity coefficients calculated for possible pairwise accessions varied from 0.08 (A65 and G8, N4-1 and G8) to 0.89 (N3 and N156), with a mean 0.32. The dendrogram inferred from UPGMA cluster analysis based on the Jaccard’s similarity matrix was shown in Figure 2. The cophenetic value was calculated 0.88, indicative of a good fit between this dendrogram and the original similarity matrix. At the Jaccard’s similarity coefficient 0.29 the dendrogram could be clustered in six major groups. Group i included the six N-coded accessions from *Neoregelia*. Group ii consisted of the eight V-coded accessions from *Vriesea*. For the accession from *Aechmea*, group iii was made up of A6, A36, A42, A51, A54 and A50, and the six S-coded selected hybrids were assigned to group iii, and A65 separately clustered into group iv, and group v comprised the other three *Aechmea* bromeliads, A5, A38, and A39. The G-coded accessions from *Guzmania* were divided into group vi.

**Figure 3:**
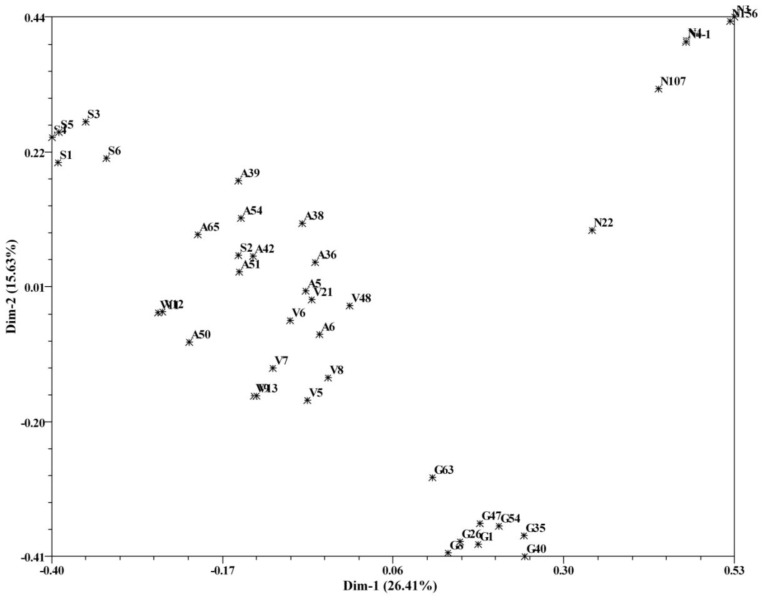
Two-dimensional matrix plot of the first two coordinates of principal coordinate analysis (PCoA), showing associations among the cultivated bromeliad accessions.

The genetic relationships among the investigated bromeliads here were also investigated using PCoA. A two-dimensional plot showing the dispersion of the 40 bromeliad accessions was displayed in Figure 3. The first two principal coordinates accounted for 26.41% and 15.63% of the total molecular variations, respectively. With respect to the two principal coordinates, accessions from *Neoregelia* and *Guzmania* were located in two distinct clusters, whereas the accessions from *Vriesea* and *Aechmea* almost dispersed together excluding the selected hybrids other than S2.

### 3.3. Bayesian-Modeled Population Structure

**Figure 4:**
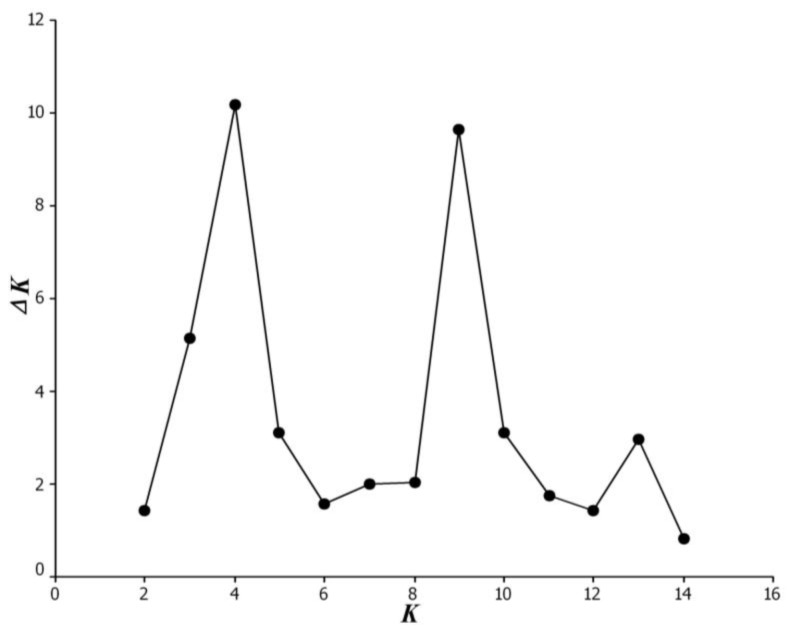
Magnitude of Δ*K* from structure analysis as a function of *K*, calculated following the Δ*K* methods as proposed by Evanno *et al.* [25], for the investigated bromeliads based on the ISSR marker data. The modal value of these distributions indicates the true *K* or the uppermost level of structure, here four subgroups.

**Figure 5:**
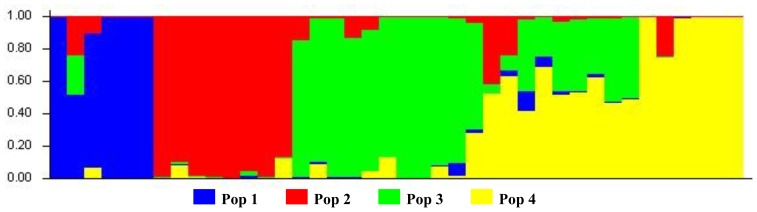
Bayesian admixture proportion of individual plants of the investigated bromeliads for a *K* = 4 population model. The *K* = 4 “subgroups”, identified by the program STRUCTURE, are indicated in different colors.

The L(*K*) achieved by STRUCTURE did not show a clear cutoff for the true subgroups with *K* ranging from 1 to 15 (data not shown), and thus the *ad hoc* measure Δ*K* was used to infer the subgroups. For the entire bromeliad accession herein, an obvious optimum regarding Δ*K* was observed at *K* = 4 (Figure 4). Based on a membership probability threshold 0.80, the eight *Guzmania* accessions were assigned to Pop 1, and the ten *Vriesea* accessions to Pop 2, the six *Neoregelia* accessions other than N22 to Pop 3, and the selected hybrids of A50 to Pop 4 excluding S2 (Figure 5). Unexpectedly, the remaining 12 accessions including the majority of *Aechmea* bromeliads were retained in a mixed subgroup due to their membership probabilities < 0.80 in any given subgroup.

## 4. Discussion

Cultivated bromeliads are of great economic importance in the flower industry and many bromeliad varieties have been bred overseas; however, little information is available concerning genetic characterization or breeding experience for cultivated bromeliads. In China, relatively few varieties of bromeliads have been bred during the last decade. This can be explained by the absence of knowledge about genetic diversity and the relationship among the collected bromeliads, together with the lack of breeding materials and poor know-how concerning breeding new varieties. Thus, a complete knowledge of genetic variations would permit future rational management and improvements of cultivated bromeliads for bromeliad breeders. However, previous studies mainly targeted the wild species of bromeliads [4–12], therefore affording a poor data base for the breeding of cultivated bromeliads. Generally, bromeliads take 2–4 years for the first natural blooming. Moreover, the performance of morphological traits is easily influenced by environmental factors and developmental stages, and thus often does not reflect the real diversity and relationship of the germplasm; whereas DNA markers are more informative and independent of those factors. In the present study, therefore, the molecular characterization of a selected collection of cultivated bromeliads, with an emphasis on genetic diversity and population structure, was explored for the first time with ISSR markers.

Many reports have shown the informativeness in polymorphism for ISSR marker in a varying range [26–29]. In this study, of the 342 bands generated by 12 ISSR primers, 287 (~84%) were polymorphic. This was lower than a recent attempt using SRAP markers [30]. The large number of polymorphic bands, together with the ISSR-based Jaccard’s similarity ranging from 0.08 to 0.89, indicated the abundant genetic diversity amongst the cultivated bromeliads investigated here. The ISSR marker is proven to be proficient for identification of hybrids [31–34] and bud sports [35–37]. Here, the bud sport N4-1 clustered with its parental line N4 (“Flandria”) but they were distinctly identified from each other with ISSR marker. The selected hybrids (S1 to S6) grouped together and also could be differentiated from the seed parent A50 (Figure 1). The above findings confirmed the reliability of ISSR marker for discriminating cultivated bromeliads.

Information about genetic diversity and population structure will be essential to provide insights into the breeding history and genetic relationship of crop germplasm. Population structure has been reported in natural populations of *Alcantarea* [6,7], *Vriesea gigantea* [9,10], and *Pitcairnia geyskesii* [38,39], but little could be found for cultivated bromeliads. In this study, three different methods, UPGMA clustering, PCoA, and Bayesian-based approach, were used to determine the level and pattern of genetic diversity and population structure present in the cultivated bromeliads based on ISSR markers. As a result, the three methods adopted here roughly reveal a similar level of population structure. The accessions from *Neoregelia*, *Guzmania*, and *Vriesea* separately clustered together with no suspense, well accordant with their botanical classifications. Along with the unequivocal definition of these genera, the higher similarity observed in the recently selected hybrids derived from a same cross [Electronic Supplementary Material] probably implies the neutrality of ISSR markers. To our knowledge, bromeliads of these three genera were the most commercialized compared to that of other types. Also with the help of registered varieties [40], we concluded that the hybridization and selection breeding in the bromeliads of these types has been more intensive during recent decades, underscoring the intense germplasm introgression and consequent close affinities among the cultivated bromeliads of *Neoregelia*, *Guzmania*, and *Vriesea*, respectively.

As for the accessions of *Aechmea*, the UPGMA clustering unexpectedly failed to cluster the *Aechmea* bromeliads together, and also the Bayesian-based structuring retained the *Aechmea* accessions apart from the five selected hybrids in a mixed subgroup, therefore suggesting a distant relationship within the cultivated bromeliads of *Aechmea*. This might be attributed to the polyphyly of *Aechmea* as reported by Sass and Specht [5]. Meanwhile, the UPGMA cluster analysis as well as PCoA demonstrated that *Aechmea* bromeliads clustered closely with *Vriesea* bromeliads, possibly indicative of their close relationship. In addition, results of PCoA showed that the first two coordinates that accounted for ~42% of the total molecular variations did not separate the accessions into distinct groups. This is probably due to the few ISSR primers successfully amplified by the robust annealing temperature, 55 °C.

It should be noted that the Δ*K* value at *K* = 9 was close to that of *K* = 4 here (Figure 5). We then compared the subgroups of the two types, and consequently found that the former revealed similar subgroups—as has already been described in the present study—apart from re-dividing some accessions retained in mixed subgroups to additional subgroups. We therefore concluded the reliability of the population structure present in the investigated bromeliads reported here.

Many studies have reported the intra-varietal genetic variability [41–43]. However, only the genotypic variations among cultivated bromeliads were investigated, with the genetic variability within asexual clones of genotypes neglected in this study. In a future study, we will attempt to investigate intra-varietal variability of cultivated bromeliads. Crossing parents with different clusters or with an inferred distant relationship will broaden the variations in next generations and therefore permit selection of excellent hybrid plants.

## 5. Conclusions

To sum up, results from this study proved the ISSR marker to be powerful for discriminating cultivated bromeliads. The ISSR marker data revealed high genetic diversity within the cultivated bromeliads investigated herein. Generally, the Bayesian-based structure analysis unraveled similar clusters with UPGMA clustering and PCoA, apart from the difference with respect to *Aechmea* accessions. The genetic diversity and population structure revealed in the present study will be beneficial for the efficient utilization of cultivated bromeliad germplasm, as well as genetic improvements and dissection of horticulturally important traits of cultivated bromeliads in the future.

## Supplementary Material



## Figures and Tables

**Table 1:** The cultivated bromeliad accessions investigated in this study.

No.	Sample Code	Name	Botanical Classification
1	N3	“Picolo”	*Neoregelia*
2	N22	“Herb Hill”	*N. ampullacea*
3	N107	“Orange”	*Neoregelia*
4	N156	“Burnsie”s Spiral”	*N. carolinae f. tricolor*
5	N4	“Flandria”	*N. carolinae*
6	N4-1	Sport of “Flandria”	*N. carolinae*
7	G1	“Diana”	*Guzmania*
8	G8	“Chloe”	*Guzmania*
9	G26	“Scarlet Star”	*Guzmania*
10	G35	“Torch”	*G. lingulata × G. conifera*
11	G40	“Clementina”	*G. lingulata* var. *minor*
12	G47	“Pink”	*Guzmania*
13	G54	“Red”	*Guzmania*
14	G63	“Caitlin”	*Guzmania*
15	V5	“Carly”	*Vriesea*
16	V6	“Barbara”	*Vriesea*
17	V7	“Kallisto”	*Vriesea*
18	V8	“Tiffany”	*Vriesea*
19	V9	“Shannon”	*Vriesea*
20	V11	“Kida”	*Vriesea*
21	V12	“Christiane”	*Vriesea*
22	V13	“Darmo”	*Vriesea*
23	V21	“Annie”	*Vriesea*
24	V48	“Select Red”	*Vriesea*
25	A5	Unknown	*Aechmea*
26	A6	“Fireman Sam”	*A. dealbata*
27	A36	“Bert”	*A. orlandiana × A. fosteriana*
28	A38	“Friederike”	*A. chantinii × A. fasciata*
29	A39	“Marqarita L.”	*A. dealbata × A. fasciata*
30	A42	Selected hybrid	*A. distichantha × A. caudata*
31	A50	Species	*A. gamosepala*
32	A51	“Ilha Grande”	*A. gracilis*
33	A54	“Lucky Stripes”	*A. gamosepala*
34	A65	“Red flamingo”	*A. fendler × A. chantinii*
35	S1	Selected hybrid	A50 *× A. recurvata* var. *recurvata*
36	S2	Selected hybrid	A50 *× A. recurvata* var. *recurvata*
37	S3	Selected hybrid	A50 *× A. recurvata* var. *recurvata*
38	S4	Selected hybrid	A50 *× A. recurvata* var. *recurvata*
39	S5	Selected hybrid	A50 *× A. recurvata* var. *recurvata*
40	S6	Selected hybrid	A50 *× A. recurvata* var. *recurvata*

**Table 2:** ISSR primers used in this study, together with the amplified results as number of total bands (TB), number of polymorphic bands (PB), % of polymorphic bands (PPB), and polymorphism information content (PIC) values.

Primer	Sequence (5′ → 3′)	TB	PB	PPB	PIC
U808	(AG)_8_C	29	23	79.31	0.31
U809	(AG)_8_G	24	19	79.17	0.26
U810	(GA)_8_T	31	26	83.87	0.32
U812	(GA)_8_A	18	17	94.44	0.34
U815	(CT)_8_G	36	29	80.56	0.26
U834	(AG)_8_YT	23	18	78.26	0.27
U840	(GA)_8_YT	39	33	84.62	0.27
U844	(CT)_8_RC	25	21	84.00	0.35
U848	(CA)_8_RG	26	25	96.15	0.32
U873	(GACA)_4_	36	34	94.44	0.28
U880	G(GA)_2_G(GA)_2_G(GA)_2_	28	19	67.86	0.37
U892	TAGATCTGATATCTGAATTCCC	27	23	85.19	0.28
Average	-	28.50	23.92	83.92	0.30
Total	-	342	287	-	-
